# Ground and In-Flight Calibration of the OSIRIS-REx Camera Suite

**DOI:** 10.1007/s11214-019-0626-6

**Published:** 2020-01-23

**Authors:** D. R. Golish, C. Drouet d’Aubigny, B. Rizk, D. N. DellaGiustina, P. H. Smith, K. Becker, N. Shultz, T. Stone, M. K. Barker, E. Mazarico, E. Tatsumi, R. W. Gaskell, L. Harrison, C. Merrill, C. Fellows, B. Williams, S. O’Dougherty, M. Whiteley, J. Hancock, B. E. Clark, C. W. Hergenrother, D. S. Lauretta

**Affiliations:** 1 Lunar and Planetary Laboratory, University of Arizona, Tucson, AZ USA; 2 United States Geological Survey Astrogeology Science Center, Flagstaff, AZ USA; 3 Solar System Exploration Division, NASA Goddard Space Flight Center, Greenbelt, MD USA; 4 Department of Earth and Planetary Science, The University of Tokyo, Tokyo, Japan; 5 Planetary Science Institute, Tucson, AZ USA; 6 Space Dynamics Laboratory, Utah State University, Logan, UT USA; 7 Department of Physics and Astronomy, Ithaca College, Ithaca, NY USA

**Keywords:** Instrumentation, Data reduction techniques, Asteroids, OSIRIS-REx, (101955) Bennu

## Abstract

The OSIRIS-REx Camera Suite (OCAMS) onboard the OSIRIS-REx spacecraft is used to study the shape and surface of the mission’s target, asteroid (101955) Bennu, in support of the selection of a sampling site. We present calibration methods and results for the three OCAMS cameras—MapCam, PolyCam, and SamCam—using data from pre-flight and in-flight calibration campaigns. Pre-flight calibrations established a baseline for a variety of camera properties, including bias and dark behavior, flat fields, stray light, and radiometric calibration. In-flight activities updated these calibrations where possible, allowing us to confidently measure Bennu’s surface. Accurate calibration is critical not only for establishing a global understanding of Bennu, but also for enabling analyses of potential sampling locations and for providing scientific context for the returned sample.

## Introduction

**Fig. 1:**
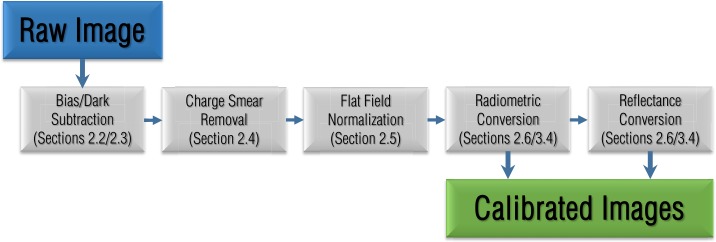
A schematic of the OCAMS image calibration pipeline that processes images from their raw form to products that are ready for scientific analysis. Each step includes a reference to the relevant section of this document

The goal of the Origins, Spectral Interpretation, Resource Identification, and Security-Regolith Explorer (OSIRIS-REx) is to study asteroid (101955) Bennu and return a sample of its surface material to Earth. OSIRIS-REx launched in September 2016 and arrived at Bennu in late 2018. The OSIRIS-REx team chose Bennu as its target because it is an accessible, slow-rotating, primitive carbonaceous asteroid, representative of the composition of the early Solar System (Lauretta et al. 2015). In the past, mission planners have divided the exploration of new planetary bodies into multiple missions with increasingly complex goals, e.g., flybys, orbits, landing, and sample return. The OSIRIS-REx mission compresses these goals into a single mission, demanding rapid observation and data analysis (Lauretta et al. 2017). This accelerated pace required that the instruments were well understood and calibrated before the encounter with Bennu, such that the data production schedule was minimally impacted by calibration activities. This manuscript reviews the analyses that contribute to the image calibration pipeline (Fig. 1) that processes every image taken by the OSIRIS-REx Camera Suite (OCAMS; Rizk et al. 2018) into science-ready products in units of radiance and reflectance. We also focus on calibration efforts that directly impact data products required by the mission to support sample site selection, such as global basemaps and color ratio maps (DellaGiustina et al. 2018).

### The OSIRIS-REx Camera Suite

OSIRIS-REx will characterize asteroid Bennu and document the sampling event with a series of instruments (Lauretta et al. 2017), including OCAMS, a triplet of optical wavelength framing cameras (Rizk et al. 2018). OCAMS includes PolyCam, a narrow-angle panchromatic imager used to create global and site-specific mosaics of Bennu’s surface; MapCam, a medium-angle imager with a series of chromatic filters to create color mosaics of Bennu’s surface; and SamCam, a moderately wide-angle imager used to document the sampling event. OCAMS has a broad range of requirements driven by the mission design as fully described in Rizk et al. (2018). Here we briefly summarize the camera properties, including details that are pertinent to calibration.

PolyCam is a 200-mm aperture diameter Ritchey-Chretien telescope, with an effective aperture diameter of 175 mm, due to obstruction by the secondary mirror and supports. The telescope has a $0.8^{\circ}$ field of view and a plate scale of approximately 13.5 μrad/pixel (the plate scale changes slightly with focus). PolyCam includes a two-lens field corrector and focus mechanism that enables it to acquire the asteroid as a point source from 1 million km away and to image the surface with sub-cm resolution from 200 m. High-resolution mapping (DellaGiustina et al. 2018) requires well-understood distortion and images that are corrected for radiometric noise.

MapCam is a 38-mm aperture diameter, five-element refractive system, with a $4^{\circ}$ field of view and a plate scale of 68 μrad/pixel. MapCam’s eight-element filter wheel allows it to image Bennu in four narrow-band color filters and a panchromatic filter. Two filter positions are used for Sun-blocking and one for refocusing the panchromatic filter at 30 m. The color filters are used to image Bennu at ranges from infinity to 500 m; the panchromatic filter retains focus as close as 125 m (or 30 m, with the alternate filter). Though radiometric accuracy is important for all three cameras, it is particularly important for the color imaging. Color ratio maps (DellaGiustina et al. 2018) highlight compositional differences on the surface and are sensitive to the relative filter-to-filter radiometric calibration.

SamCam is a 4.3-mm aperture diameter, five-element refractive system, with a $20.8^{\circ}$ field of view and a plate scale of 349 μrad/pixel (this updated value replaces the value given in Rizk et al. 2018). SamCam has a six-element filter wheel; the passbands of the filters are identical, but allow for imaging during multiple sampling attempts, should the filters become dust-covered during an attempt. One of the elements is also a diopter that refocuses SamCam from its nominal 5-m focus to 2 m, for imaging the sampler head after a collection attempt.

Each OCAMS imager was built with the same Teledyne DALSA charge-coupled device (CCD) detector, which allows the detector performance to be analyzed in consistent ways (Rizk et al. 2018).

### OCAMS Detector Layout

**Fig. 2:**
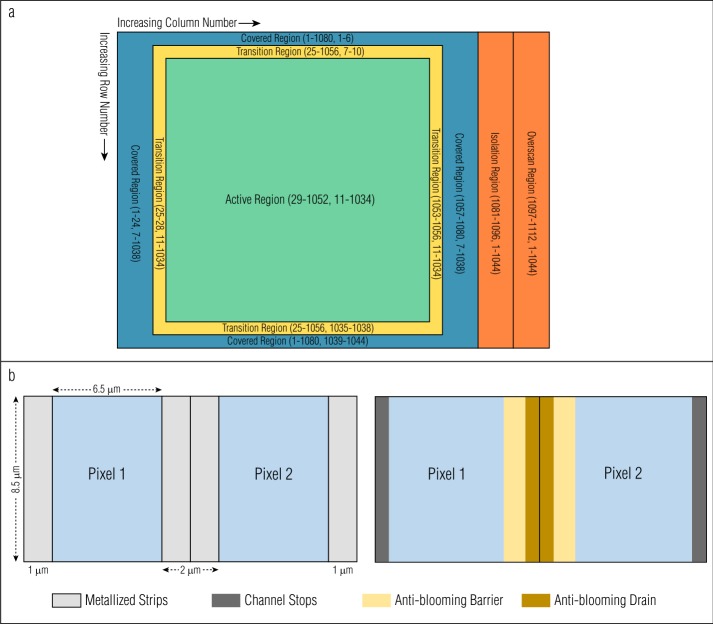
(**a**) OCAMS detector region layout, showing (column, row) extents. (**b**) Opaque metalized strips (light gray), which overlay the edges of every pixel (blue), increase the detector transfer speed, but reduce the optically sensitive area to a $6.5 \times 8.5$ μm region. Anti-blooming barriers (yellow), bisected by anti-blooming drains (brown), are located beneath the surface between every pair of pixels. Channel stops (dark gray) prevent charge from transferring between pairs of pixels

Figure 2(a) shows a schematic of the OCAMS CCD layout, with the (column, row) ranges for each region specified. The column/row indexing shown here and throughout this manuscript is 1-based, i.e., the center of the first pixel in the array is at $(1,1)$. The location of the origin (in this case, the top-left) is arbitrary and not universal. Many image viewers and data analysis programs place the origin in the bottom-left, leading to an image that appears vertically flipped. Images throughout this manuscript are displayed with their origin in the top-left. Describing detector and image locations in terms of ‘top’ and ‘bottom’ corresponds to the readout-adjacent and readout-distant rows, respectively. However, we describe all equations or calibrations in terms of row number, which is independent of image orientation.

The physical CCD is $1080 \times 1044$ pixels, with pixels spaced on an $8.5 \times 8.5$ μm grid. As shown in Fig. 2(b), each pair of pixels has sub-surface anti-blooming barriers and an associated drain that prevent charge from overflowing into neighboring pixels. In addition, vertical metalized strips overlay the boundaries between pixel columns; these strips lay on top of the anti-blooming drains and the channel stops. These strips enhance the readout speed of the detector and decrease the asymmetry of the buried structures. These regions are optically opaque, such that the optically sensitive regions of the pixels are $6.5 \times 8.5$ μm. All radiometric calibrations (Sects. 2.6 and 3.4) implicitly include the size of the pixels, so no adjustment is necessary for their fill factor. However, the optically opaque regions partially mask the detection of point sources (Sect. 3.3).

Figure 2(a) also depicts the detector’s pixel regions. The center $1024 \times 1024$ pixels are the *active region*, i.e., the region of the detector that is exposed to light when acquiring an image. A calibrated OCAMS image is $1024 \times 1024$ pixels. The outside border of pixels is the *covered region*, from which light is physically blocked, even with the camera shutter open. This region is 24 pixels (columns) wide on the left and right sides, and 6 pixels (rows) tall on the top and bottom sides. Although these pixels are masked from sensing optical light, they detect the dark (thermal) signal of the detector during an exposure (Sect. 2.3). The four pixels (rows or columns) between the active and covered regions are the *transition region*, which serves as a buffer between the two, to prevent any light that leaks under the cover from being measured in the covered region. The transition regions are not used in OCAMS calibrations and are not included in calibrated images.

The readout electronics process OCAMS images one row at a time (Sect. 1.3). At the end of each row of physical pixels, the electronics perform 32 *empty reads* of the readout buffer. These reads do not correspond to physical pixels and will not represent any optically or thermally generated electrons. We can therefore use them to evaluate the bias level and read noise of the detector (Sect. 2.2). The 32 empty reads create a 32-column region on the right side of the image (because they are read out after each physical row is read). The first 16 columns of this region are the *isolation region*, and are typically ignored to allow any residual signal in the readout register to subside. The final 16 columns are the *overscan region* and are used for bias calculations.

### OCAMS Image Acquisition

**Fig. 3:**
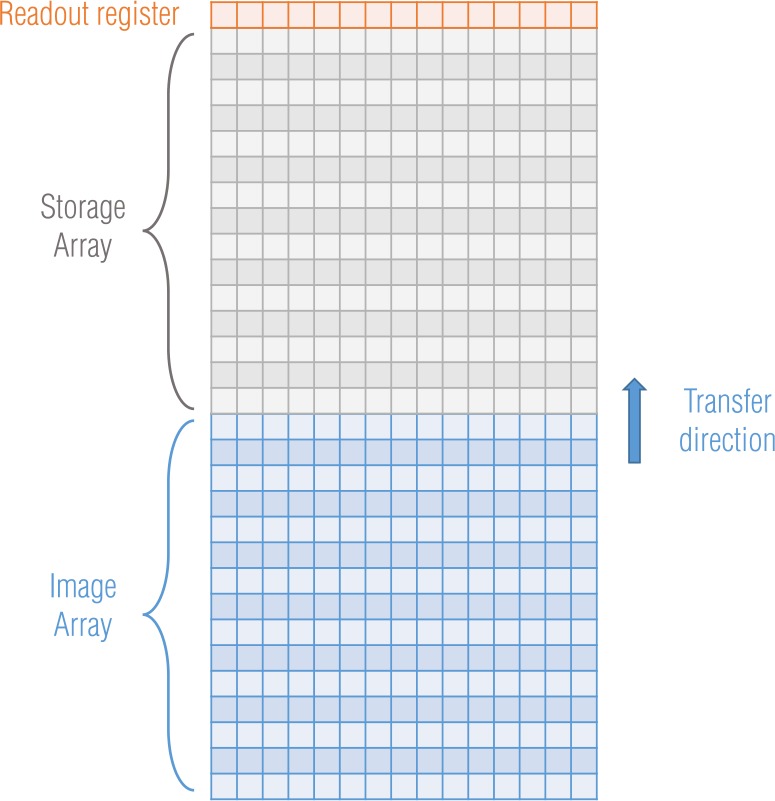
OCAMS CCD frame transfer architecture

The OCAMS detectors are frame transfer CCDs, for which the image array is transferred to a covered (dark) storage array (Fig. 3). Once in the storage array, the rows are read out, one at a time, via the serial readout register. The transfer direction is toward decreasing row number (i.e., row 1 is read out first and row 1044 is read out last). The details of the readout process, which we describe in this section, are critical to a number of calibration processes, including radiometric conversion, which depends on precise knowledge of exposure times (Sects. 2.6 and 3.4), and charge smear correction, which depends on the way in which pixels are transferred during exposure (Sect. 2.4).

The readout register can transfer the pixels in *left-tap* or *right-tap* mode, which are functionally redundant, identical, electronic paths on the left and right sides of the detector. The CCD can also read the pixels out in split- (or dual-) tap, which uses both taps to increase readout speed. During ground testing and in flight, the cameras’ default readout mode has been left-tap, but the images are flipped horizontally when they are stored in the OSIRIS-REx ground system data processing infrastructure (Selznick 2017), as if they were taken in right-tap mode. This format presents images in the same orientation as an observer located at the camera position and maintains consistency with images from ground testing. We acquired all images in this manuscript in left-tap mode but present them with the horizontal flip. Because they are not physical pixels, the isolation and overscan regions remain on the right side of the image in right-tap mode. Therefore, we flip only the physical pixels (columns 1–1080) horizontally in a left-tap image to make it appear right-tap.

The CCD electronics transfer the pixels from the image array to the storage array at a rate of one row per μs, such that the full array takes 1.044 ms. Subsequent transfer of all rows through the readout register takes an additional 0.2322 ms. The readout register is periodically flushed during an exposure to remove any old signal that has accumulated in the register; flushing the readout register takes 0.285275 ms. The commanded exposure time includes frame transfer time, but not the readout register flushing time. Therefore, a 10-ms image involves 8.956 ms of static exposure (including 0.285275 ms for flushing the serial readout register) and 1.044 ms of frame transfer (during which optically and thermally generated signal is still being accumulated; Sect. 2.4).

Frame transfer must still occur for very short exposures. Commanding a 0-ms exposure does not produce a 0-ms image, as the CCD must transfer the pixels on and off the chip. The total exposure time for a 0-ms command is 1.494075 ms, of which 0.450075 ms are static exposure to the scene. For longer exposure times, this offset is a fixed 0.285275 ms. Table 1 shows the list of exposure times, where *total exposure time* includes the frame transfer and flushing times, and *effective exposure time* indicates the time during which the static pixel array accumulates charge. The difference between these two exposure times will be critical for charge smear correction and subsequent radiometric conversion (Sects. 2.4.2 and 2.6). Here and throughout this document, the exposure times given are those recorded in the headers of OCAMS images, at the precision of the FPGA clock which controls them.

**Table 1:** OCAMS effective exposure time

Commanded exposure time (ms)	Total exposure time, single tap (ms)	Total exposure time, split tap (ms)	Effective exposure time (ms)
0	1.494075	1.271675	0.450075
1	1.494075	1.271675	0.450075
2	2.554475	2.220875	1.510475
3	3.224675	3.113475	2.180675
≥4	Commanded + 0.285275	Commanded + 0.113475	Commanded + 0.285275–1.044

**Fig. 4:**
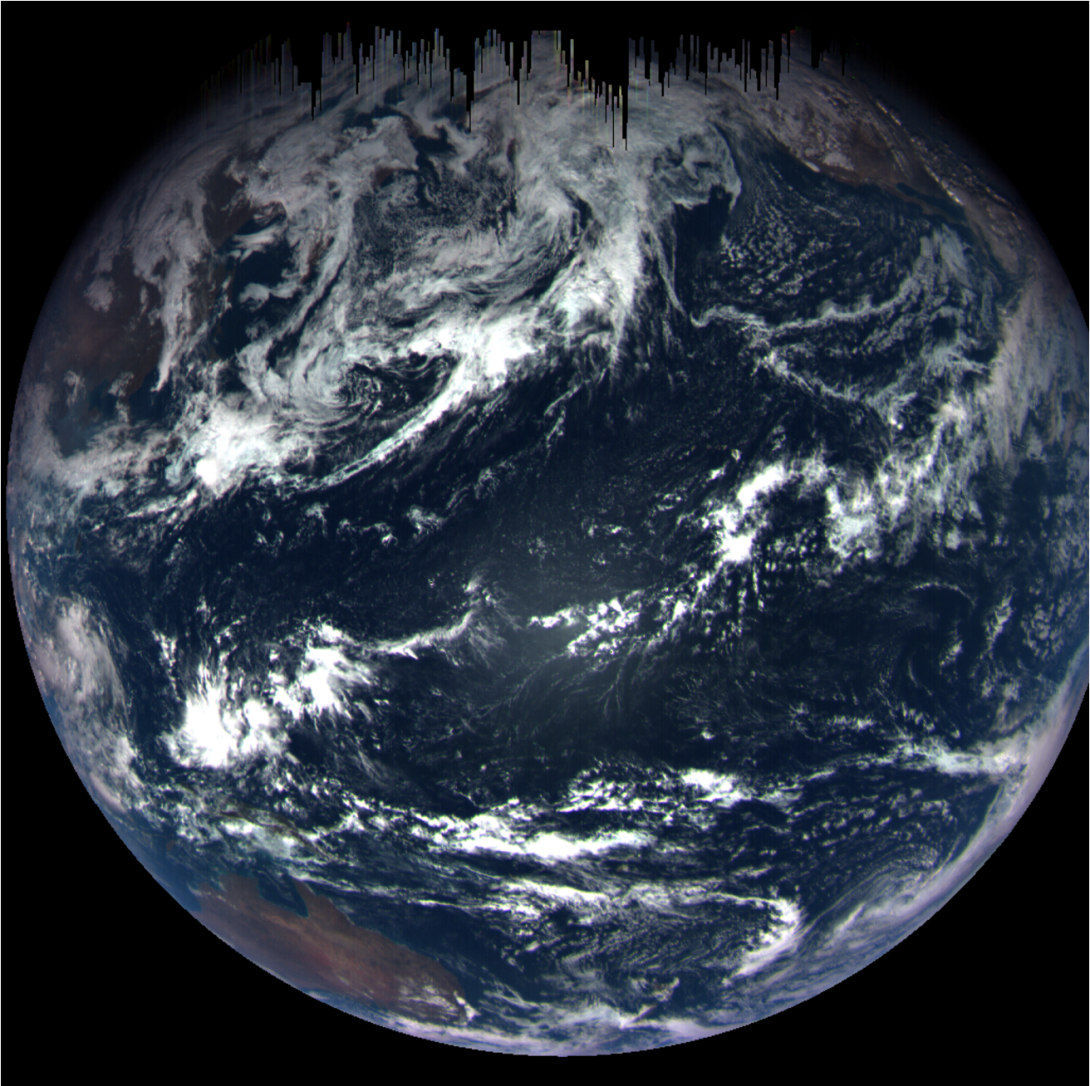
Color portrait of Earth acquired during OSIRIS-REx’s EGA maneuver in September 2017, which put the spacecraft on its trajectory to reach Bennu. Observations of Earth and the Moon were collected during this maneuver to check and calibrate the instruments. Earth was such a bright target that it required static exposure times of 0.45 ms, precluding the possibility of a final storage area flush and horizontal serial register flush. The incident light overwhelmed the serial registers with signal, which overflowed onto the image in the storage area, shown here as icicles at the top (the location of the serial register in this image). The length of each contaminating line is a reflection of the accumulated brightness in the column below it

When commanded to take an image, the electronics clear the CCD storage area of charge in a final pre-flush before transferring active, exposed charge into it. This process, which includes flushing the 1044 rows of the storage section into the serial register and dumping the charge from the register, can take over 2 ms, depending upon the exact phasing with respect to the next millisecond timing pulse. For most exposure times, ∼2 ms is less than the static time corresponding to commanded exposure time, thus a final flush to clear the storage area and horizontal readout register is possible for all exposure times of 4 ms or greater. However, for commanded exposure times of 2, 1, or 0 ms, the static time is shorter than the flushing process. Even for a commanded exposure time of 3 ms, the timing is close enough that some residual signal can remain. In these circumstances, the electronics read out the image without a complete final flush. This typically causes partial image corruption because the storage section is not completely cleared of charge and thus the serial register can be overfilled during the final frame transfer. The extraneous charge will transfer back into the storage section and be read out as signal. The amount of charge is often large because any short-exposure image is likely due to a very bright scene, thus a considerable amount of charge has accumulated before the exposure. This effect occurred during the MapCam three-band color portrait of Earth, shown in Fig. 4, during the OSIRIS-REx spacecraft’s Earth gravity assist (EGA) (Golish et al. 2018). In the original images, the overflowing signal presents as vertical lines of saturated pixels, colloquially called *icicles*; here we have masked out the icicle regions. Images of Bennu, which is a much darker object, exhibit this effect less often, though images at very low phase angles and near perihelion still require exposure times under 4 ms.

### Data Availability

We archive all OCAMS data in the publicly accessible Planetary Data System (PDS) Small Bodies Node (Rizk et al. 2019). OCAMS raw and calibrated images, as well as calibration files, are delivered to the PDS according to the OSIRIS-REx Data Management Plan (Crombie et al. 2018) available in the OSIRIS-REx PDS archive (https://sbn.psi.edu/pds/resource/orex/). Ground test data used in the calibration effort will also be archived in the PDS Small Bodies Node.

## Ground Calibrations

The OCAMS cameras went through rigorous ground tests designed to validate and characterize their performance and to enable the development of pre-flight calibrations. Rizk et al. (2018) discusses the optical validation and performance aspects in detail. Calibration activities included characterization, for all three cameras, of the bias level, dark current rates, flat field response, and radiometric conversions. The impact of temperature variation on these attributes was also included where applicable. This section describes the image calibration steps that correct these effects.

### Bad Pixels

During ground testing, we identified *bad pixels*—pixels with abnormal sensitivity—that we group into three categories. *Hot pixels* are highly sensitive to thermally generated current and have dark current generation rates 10 to 10000 times greater than the bulk of the detector. These pixels are few in number (≪1% of the array) and only affect long-exposure images (>100 ms). We also monitored the detectors for *dead pixels*, which are less sensitive than a typical pixel, but observed none in ground testing. The third population, called *random telegraph signal* (*RTS*) *pixels* (or *flicker pixels*), exhibits a temporally varying behavior. These pixels change state between hot and normal (or sometimes between multiple levels of sensitivity). RTS pixels vary with temperature, time, and exposure length, making them impossible to characterize in a universal map. At short exposure times (<100 ms, which is the vast majority of OCAMS imaging), a small number (∼30) of RTS pixels are measurable. For very long exposures (e.g. 20 seconds, typically acquired to image star fields), the population rises to as high as 3% of the total number of pixels.

We have developed a historical bad pixel map that identifies this population of pixels. Because bad pixel correction criteria and methods are often specific to the type of scientific application, the OCAMS calibration pipeline does not automatically correct images for bad pixels when it calibrates and converts the images into radiometric units. However, the PDS archive of the image data includes historical and per-image bad pixel maps (Rizk et al. 2019).

The calibration pipeline identifies bad pixels per image by finding pixels that are statistically different from their neighbors. Though the parameters of the search are configurable, by default the calibration pipeline analyzes a $10\times10$-pixel window that the pipeline sweeps across the detector in five-pixel steps. Any pixels within the window that are more than five sigma larger or smaller than the mean of that window are marked as bad (hot or dead, respectively).

### Bias

The OCAMS detectors, as with most CCDs, add a bias offset to the output of the analog-to-digital converters that translate the number of electrons measured by the detector to digital numbers (DN) recorded by the electronics (Janesick 2001). This offset prevents the output, which has random read noise that causes the signal to fluctuate around its mean, from dropping below zero when low-signal images are converted to DN (an unsigned 14-bit integer). Bias level varies slightly with detector temperature and the level of optically generated signal.

Each CCD possesses an individual bias level per readout direction (left-, right-, or split-tap; Sect. 1.3). Accordingly, the bias behavior was measured for each camera and readout combination during ground testing by taking bias images. Bias images are dark (i.e., taken with a light blocking filter in place) and have the minimum exposure time possible. As discussed in Sect. 1.3, even a 0-ms commanded exposure has a ∼1.5-ms exposure time, thus measuring a true bias is not possible. However, for a dark image, with the low dark current in these detectors (Sect. 2.3), the non-bias component of a bias image is minor.

**Fig. 5:**
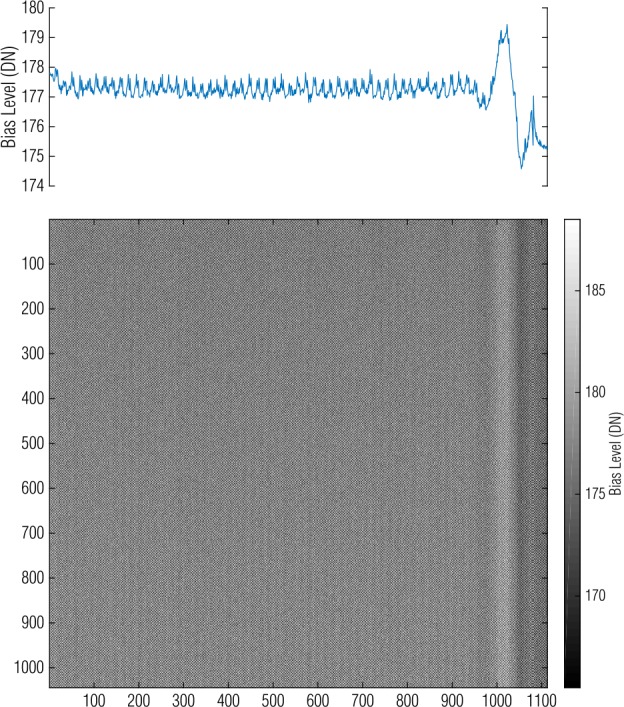
A MapCam master bias image depicts columnar spatial variation. The plot at the top represents the average of each column and depicts the sinusoidal roll-on effect on the right side

We measure the bias level by taking many bias images and calculating their mean to form a *master bias*. The mean signal in the master bias measures the bias level set by the electronics. The standard deviation of the pixel signals is a measure of the read noise in the electronics. For the OCAMS detectors, the read noise is on the order of 10 DN (Rizk et al. 2018). The master bias represents the bias behavior at the time of acquisition. A master bias for MapCam is shown in Fig. 5 and is representative of the other OCAMS detectors. As expected, the majority of the image appears as random signal, but there is a distinct feature on the right side. This sinusoidal variation in bias level in the row direction, referred to here as the *roll-on effect*, appears in all images and has a magnitude of ∼6 DN. It occurs for the first ∼100 pixels that are read out (these images are left-tap, but displayed as right-tap, so the readout direction is to the right). This effect appears to be an artifact of the electrical system that controls the CCD, but its exact cause is unknown. Nonetheless, by using a master bias, we account for this feature in our image calibration; any non-spatial correction (i.e., subtracting a single mean bias value off all pixels) would result in residual structure.

Bias level varies slightly with temperature, approximately 0.1 DN/$^{\circ}\mbox{C}$, suggesting that a master bias will be more accurate if taken at the same temperature as (or concurrently with) the data that it corrects. In addition, the bias level varies from image to image by approximately 1 DN. This prompts the use of on-chip methods to correct the bias as well. However, correcting for bias independently from dark current requires additional operations, which introduce small amounts of processing noise (Newberry 1991). Instead, the calibration pipeline corrects bias in tandem with dark current.

### Dark

**Fig. 6:**
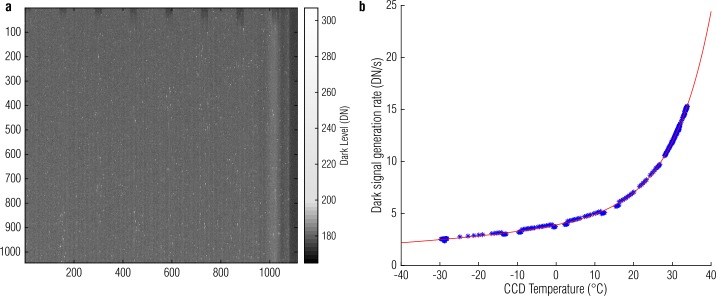
A MapCam master bias/dark image, shown with a non-linear stretch that visualizes both the bulk signal level and the bright hot pixels (**a**) and its temperature model (**b**)

Thermally generated electrons within the silicon produce dark current that is measured as electronic signal (Janesick 2001). The dark current generation rate in the OCAMS detectors is low but non-negligible, especially for long-exposure images (e.g., star field observations). We measure the dark current in a similar manner to the bias level, by calculating the mean of many images at a given exposure time and temperature (dark current is temperature dependent) to create a *master dark*, which represents the dark current for every pixel. Like bias, these images are most accurate when taken concurrently (e.g., just before or after) the images that they will correct. A master dark inherently includes the bias level. Therefore, there is no reason to calculate a master bias separately; we can correct both effects with a single master. Figure 6(a) depicts a 500-ms MapCam master dark. The mean dark current contribution is approximately 5 DN, but hot pixels have signals as high as 50 DN above the bias level. The columnar effects of the bias level are still visible.

Dark current is strongly temperature-dependent; more electrons are thermally generated when the silicon is warmer. Though all flight observations include concurrent dark images (taken at approximately the same temperature), ground calibration activities explicitly measured this dependence. Figure 6(b) shows this relationship for MapCam, which we express as a sum of two exponentials; this formulation is empirical and not representative of an underlying physical effect. Though temperature dependence is typically described as a single exponential (Janesick 2001), we find that it does not describe these CCDs well. In particular, these detectors have two regimes, below and above ${\sim} {+}10\ ^{\circ}\mbox{C}$, that are better described with a sum of exponentials.

$$ R_{dark} =a* e^{b*T} +c* e^{d*T} $$

where $R _{\mathit{dark}}$ is the dark signal generation rate (DN/s), $T$ is the CCD temperature ($^{\circ}\mbox{C}$), and the other variables are model fit parameters. The parameters for MapCam, PolyCam, and SamCam are listed in Table 2.

**Table 2:** Dark model parameters

Camera	a	b	c	d
MapCam	3.59	0.0125	0.312	0.102
PolyCam	2.47	0.0148	0.295	0.101
SamCam	3.07	0.0143	0.299	0.100

The OCAMS calibration pipeline corrects bias and dark together (referred to as bias/dark) in two steps. The first subtracts the master bias/dark array directly off the image. The result is an intermediate image that is largely free of both effects, but small instantaneous variations in bias level may still exist. To compensate for these variations, the calibration pipeline examines the covered column regions of the CCD. These regions should contain only bias and dark current signal, as they are masked from light (Sect. 1.2). If correction via the master bias/dark were perfect, these regions would have a mean signal of zero (with read and shot noise variation around that mean). To the extent that the bias or dark has drifted (which is largely driven by the image-to-image bias variation mentioned in Sect. 2.2), the covered columns will capture its magnitude. However, because the covered columns are physical pixels, they are also susceptible to both hot pixels and cosmic ray hits. To prevent their contribution to the correction, the covered columns are scrubbed for bad pixels (Sect. 2.1). The calibration pipeline corrects bad pixels by replacing their value with the mean of their four nearest neighbors. To apply the instantaneous bias/dark correction, the pipeline calculates the median of the covered columns, on a row-wise basis, to produce a column vector. That vector is boxcar-averaged with a 50-pixel kernel to smooth row-to-row variations due to small-number statistics (only 48 columns contribute to median for each row). That smoothed column vector is then subtracted off each row of the intermediate image.

$$\begin{aligned} \boldsymbol{I}_{ij} =& \boldsymbol{S}_{ij} - \boldsymbol{BD}_{ij} \\ c_{i} =&\mathit{median} \{ I_{i,1} \dots I_{i,24}, I_{i,1057} \dots I _{i,1080} \} \\ c_{i} ' =&\mathit{boxcar}( c_{i},50) \\ \boldsymbol{S}_{i} ' =& \boldsymbol{I}_{i} - c_{i} ' \end{aligned}$$

where $\boldsymbol{S} _{ij}$ is the raw signal, $\boldsymbol{BD} _{ij}$ is the master bias/dark, $\boldsymbol{I} _{ij}$ is the intermediate image, $c _{i}$ is the row-wise median of the covered columns, $c _{i} '$ is the boxcar smoothed vector, and $\boldsymbol{S} _{i} '$ is the final corrected image.

### Charge Smear

**Fig. 7:**
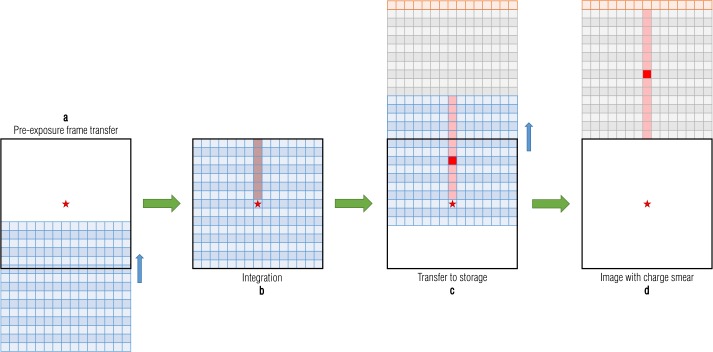
A schematic depiction of the CCD frame transfer process and resulting charge smear

Charge smear is the result of signal generated in pixels while the electronics are transferring the array in and out of the active region. The effect is most obvious when a bright source is in the field of view of the CCD, as we show in the following example. Figure 3 depicts a frame transfer CCD, which has an image array exposed to light in the bottom half (blue), a masked storage array in the top half (gray), and a readout register (orange). The process for taking an image is shown in Fig. 7; the red star represents a bright source in the scene. The process begins when the electronics transfer a new frame on to the image array (a). Any charge that had been accumulated in the image array before the exposure is transferred to the storage array and discarded. As the rows in the top portion of the image array are transferred past the star, they collect a small amount of charge, denoted by the faint red trail (b). The pixels then collect charge for the designated exposure time (modulo the frame transfer time; Sect. 1.3). After the integration timer expires, the electronics transfer the frame to the storage array; here the rows below the star are briefly exposed to light as they transfer past it (c). When the frame transfer is complete, the data in the storage array looks like (d); the star created a bright response in the center and a faint trail in both directions. The serial register reads out the image row by row. No additional optical charge is accumulated during this time (the storage area is masked), though a negligible amount of dark current is generated.

The advantage to this architecture is that all pixels are exposed for the same amount of time. Pixels “above” that source (i.e., between the corresponding pixel and the readout) are briefly illuminated as the array is transferred in, before the exposure. Pixels “below” the source are briefly illuminated as the array is transferred out. Though this transfer time is small (1.044 ms total, 1 μs per row), a sufficiently bright source will generate measurable charge in columns with pixels that the source is illuminating. Moreover, a bright source likely indicates a short exposure time, such that the transfer time is a substantial fraction of the total exposure time.

Covered rows are also subject to charge smear because they are exposed to the light as they are transferred past the location of the source. Accordingly, covered rows provide an empirical measure of charge smear, as they are representative of the signal generated during transfer (plus dark current) but contain no signal generated during the exposure. However, the small number of covered rows provide poor statistics and the resulting correction has noticeable vertical artifacts. Even smoothing the correction vector, as we do with bias/dark correction, does not eliminate these artifacts.

Alternatively, we can predict charge smear from the signal in the image. In theory, this is valid only for columns that contain no saturated pixels, as a saturated pixel does not record how bright that point truly is. However, the bulk of the albedo variation on Bennu’s surface (Lauretta et al. 2019) can be captured within the dynamic range of the OCAMS detectors, such that any saturated pixels are not far outside the dynamic range and represent a small percentage of the total population. We estimate the charge smear that should exist, per column, using only the exposure time and measured signal in the image. Unfortunately, for very short exposures that exhibit icicle artifacts (Sect. 1.3), the icicle regions must be ignored when calculating charge smear based on the total measured signal. This reduces the fidelity of the charge smear correction, because it cannot capture the signal variation within those rows.

The integrated signal recorded by the detector due to the full imaging operation (exposure time and frame transfer time) can be measured by summing the signal on a column-wise basis. This sum is the full amount of charge accumulated. A fraction of that charge (the ratio of the row transfer time to the integration time) is distributed to each pixel in the column. We cannot simply remove this fraction from every pixel in a given column, as it also includes signal generated by the charge smear. We therefore solve for the true amount of charge smear. The total signal measured by a given pixel is the combination of the true signal from its location in the scene and the charge smear from locations above and below it.

$$ \boldsymbol{S} = \hat{\boldsymbol{S}} + \boldsymbol{E} $$

where $\boldsymbol{S} $ is the total measured signal, $\hat{\boldsymbol{S}}$ is the true signal from the scene, and $\boldsymbol{E} $ is the contribution from charge smear. For pixel ($i, j$), the charge smear contribution is proportional to the sum of the actual signals in that column.

$$ \boldsymbol{S}_{i,j} = \hat{\boldsymbol{S}}_{i,j} + \epsilon \sum_{i} \hat{\boldsymbol{S}_{j}} $$

where ($i, j$) is the (row, column) of the pixel location and $\epsilon $ is the ratio of row transfer time to exposure time (i.e., what fraction of the exposure each pixel is exposed to the other locations in the scene). The signal measured in a column is then the sum of all pixels in that column.

$$ \sum_{i} \boldsymbol{S}_{j} = \sum _{i} \biggl( \hat{\boldsymbol{S}} _{i,j} +\epsilon \sum_{i} \hat{ \boldsymbol{S}_{j}} \biggr) $$

$\sum_{i} \boldsymbol{S}_{j}$ is the sum of the $j$th column in the image. It is an input to the solution, so will be represented as the variable $\boldsymbol{Y} $. We substitute this in, as well as substituting $\hat{\boldsymbol{S}} = \boldsymbol{S} - \boldsymbol{E}$.

$$ \boldsymbol{Y}_{j} = \sum_{i} \biggl( ( \boldsymbol{S}_{j} - \boldsymbol{E}_{{j}} ) + \epsilon \sum_{i} ( \boldsymbol{S}_{j} - \boldsymbol{E}_{{j}} ) \biggr) $$

Again, we substitute the measured signal in the column, $\boldsymbol{Y} $.

$$ \boldsymbol{Y}_{j} = \sum_{i} \biggl( \boldsymbol{S}_{j} - \boldsymbol{E}_{{j}} + \epsilon \boldsymbol{Y}_{j} -\epsilon \sum _{i} \boldsymbol{E}_{{j}} \biggr) $$

The sum of the charge smear term over the column is merely the charge smear term, summed $N_{\mathit{row}}$ times (the number of rows, 1044).

$$ \sum \boldsymbol{E} = N_{\mathit{row}} * \boldsymbol{E} $$

We can therefore substitute further and distribute the summing operation.

$$\begin{aligned} \boldsymbol{Y}_{j} =& \sum_{i} \boldsymbol{S}_{j} - \sum_{i} \boldsymbol{E}_{j} + \sum_{i} \epsilon \boldsymbol{Y}_{j} - N_{\mathit{row}} * \epsilon \sum _{i} \boldsymbol{E}_{{j}} \\ \boldsymbol{Y}_{j} =& \boldsymbol{Y}_{j} - N_{\mathit{row}} * \boldsymbol{E}_{ {j}} + {N}_{\mathit{row}} *\epsilon * \boldsymbol{Y} _{{j}} - {N}_{\mathit{row}}^{2} * \epsilon * \boldsymbol{E} _{{j}} \end{aligned}$$

Finally, we can solve for the charge smear term, which will convert the total measured signal of any pixel in the $j$th column to the true signal from the scene.

$$ \boldsymbol{E}_{{j}} = \frac{\epsilon \boldsymbol{Y}_{j}}{N_{\mathit{row}} \epsilon +1} $$

In practice, this approach does not completely correct charge smear. We have been unable to identify the source of this discrepancy, though we have seen that it is more exaggerated at shorter exposure times. This leads us to predict that the flaw is in our understanding of either the timing of the frame transfer process or the effect of detector linearity (Sect. 2.6.2) within it. Without a physical justification, we use the covered rows to empirically evaluate the residual and improve the correction. If the algorithm outlined above perfectly predicted charge smear, the covered rows would have a mean signal of zero (assuming bias and dark signals have already been removed). Mean signal above or below zero in the covered rows indicate under- or over-correction in the charge smear algorithm. We can therefore modify the magnitude of the correction in the appropriate direction and iterate until the mean signal in the covered row region is minimized. This method is less susceptible to the small number of covered rows, because we can calculate the median of all the columns together to improve the statistics. For most exposure times, the magnitude of this correction is on the order of 10–20%. However, for the shortest exposure times (e.g. 1 ms), the correction can be as high as 90%.

#### Guided Charge Smear

**Fig. 8:**
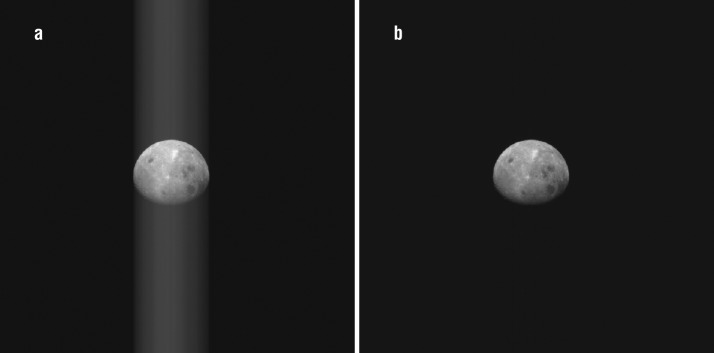
A PolyCam image of the Moon taken on 25 September 2017 before (**a**) and after (**b**) charge smear correction. The guided method reduces the charge smear by ∼99%

Images of the Moon and Earth taken during the OSIRIS-REx EGA (Golish et al. 2018) and the initial whole-disk observations of Bennu (DellaGiustina et al. 2019; Hergenrother et al. 2019; Lauretta et al. 2019) provide an opportunity to improve on-chip charge smear correction. Portions of these observations image only dark space, with no measurable signal. However, those dark pixels do transition past other locations in the scene (e.g., the Moon) and collect charge smear. Thus, they are accurate representations of the charge smear, in the same way as the covered rows, but with better statistics (as a function of the number of rows that image dark space). This method is not automatic, as it requires user guidance to define the appropriate image rows that observe dark space. Despite the manual nature of this correction, we have applied it to all whole-disk images taken by OCAMS to date. In the example shown in Fig. 8 from OSIRIS-REx’s EGA, the algorithm decreases the charge smear by ∼99%.

#### Exposure Time Correction

Removing the signal associated with charge smear requires removing the exposure time associated with frame transfer. If the exposure time is not adjusted, radiometric conversions that depend on exposure time (Sect. 2.6) will be incorrect by the ratio of frame transfer time to total exposure time. Accordingly, the calibration pipeline calculates the effective exposure time by subtracting the frame transfer time (1.044 ms) from the total exposure time, as discussed in Sect. 1.3, and records it in the FITS header.

### Flat Field

Owing to optical vignetting, shadowing, and detector fixed pattern noise, the spatial response of the detector to incoming light is not uniform. This non-uniformity can be corrected by measuring and applying a flat field (Janesick 2001). In ground testing, the OCAMS cameras observed a spatially invariant source at an exposure time that measured approximately 8000 DN. For MapCam and SamCam, the spatially invariant source was a $20''$ integrating sphere (Rizk et al. 2018). The open port of the sphere was too narrow for PolyCam, so it used an Alnitak Astrosystems Flatman-XL light panel. We calculate the mean of many such images to create a *master flat*. We normalize the master flat to its mean and invert it, such that the calibration pipeline can apply the flat in a multiplicative manner to correct the camera’s spatial variance.

$$\begin{aligned} \boldsymbol{F} ' =& \frac{\sum_{n} \boldsymbol{SI}_{n}}{n} \\ \boldsymbol{F} =& \frac{{\sum_{i=1,j=1}^{1024,1024} \boldsymbol{F}' _{ij}} / { ( 1024*1024 )}}{\boldsymbol{F}'} \end{aligned}$$

where $\boldsymbol{SI} _{n}$ is the stack of spatially invariant, bias/dark-corrected, flat field images; $n$ is the number of images; and $\boldsymbol{F} $ is the master flat.

The calibration pipeline applies the master flat by multiplying it with the image to be corrected.

$$ \boldsymbol{S} ' = \boldsymbol{S} * \boldsymbol{F} $$

where $\boldsymbol{S} $ is the uncorrected image and $\boldsymbol{S} '$ is the corrected image.

**Fig. 9:**
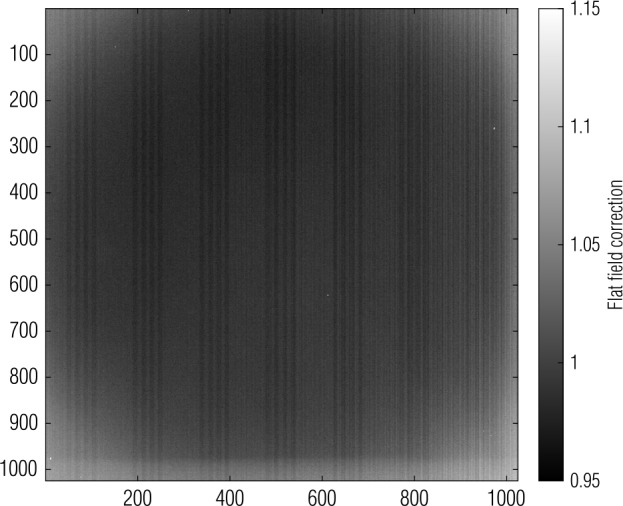
A MapCam master flat is normalized to its mean and inverted to make its application multiplicative; bright areas will be amplified when the correction is applied

Figure 9 shows the master flat for MapCam with the pan filter. Visual inspection reveals three dominant effects. Optical vignetting causes dimming in the corners (which in turn are brighter in the multiplicative flat field). Fixed pattern noise manifests as an every-other-column variation in sensitivity. The detector housing causes a partially shadowed band at the bottom edge of the image. The master flat corrects for these effects when applied to an image.

### Radiometry

The ground-based radiometric calibration of OCAMS converts the detected signal in DN to units of radiance. For the panchromatic filters, we use broadband radiance ($R$), which has units of $\mbox{W}/\mbox{m}^{2}/\mbox{sr}$. For the color filters, we use spectral radiance ($R _{\mathit{spec}}$), which has units of $\mbox{W}/\mbox{m}^{2}/\mbox{sr}/\upmu \mbox{m}$. In preflight radiance testing, as described in Rizk et al. (2018), the cameras imaged a source of known radiance. We measure the radiance presented to the cameras with a Gooch & Housego OL 730-5A detector. The detector is commercially calibrated against a Gooch & Housego NIST-traceable silicon detector such that its spectral responsivity is known from 250 to 1100 nm (the active bandpass of the detector), with a transfer uncertainty of ±0.5% (Gooch & Housego 2019). The detector measures the amount of current generated by the incoming light in the band of a desired filter, which we convert to radiance. The ratio of camera signal rate to this value is the camera’s responsivity.

We predict the current that the calibrated detector would theoretically measure in the presence of a normalized laboratory source ($E _{ \mathit{norm}}$). Because we use this current in a ratio with respect to the measured current, the intensity of the source for the predicted current is arbitrary; therefore, its peak irradiance is normalized to 1 $\mbox{W}/\mbox{m}^{2}$.

$$ I_{\mathit{norm}} = \int _{0.25~\upmu \text{m}}^{1.1~\upmu \text{m}} E_{\mathit{norm}} ( \lambda ) * r_{\mathit{det}} ( \lambda ) * T_{\mathit{filter}} ( \lambda ) d\lambda $$

where $I _{\mathit{norm}}$ is the current that would be measured by the calibrated detector, $r_{\mathit{det}}$ is the responsivity of the calibrated detector, $E _{\mathit{norm}}$ is the normalized laboratory source spectrum, and $T _{\mathit{filter}}$ is the transmission curve of the filter in use. We measure the spectrum of the laboratory source with an Ocean Optics USB4000 fiber optic spectrometer. The spectral response of the spectrometer is calibrated with an Oriel 6035 Hg(Ar) calibration lamp.

We use the ratio of predicted to measured current to scale the irradiance of a normalized source to a measured source. Applying the ratio of currents gives us the in-band irradiance of the source ($\mbox{W}/\mbox{m} ^{2}$).

$$ E_{\mathit{band}} = \frac{I_{\mathit{det}}}{I_{\mathit{norm}}} E_{\mathit{band},\mathit{norm}} $$

where $E _{\mathit{band}}$ is the measured in-band irradiance ($\mbox{W}/\mbox{m} ^{2}$), $I_{\mathit{det}}$ is the current (A) measured by the calibrated detector, and $E_{\mathit{band},\mathit{norm}}$ is the integrated irradiance ($\mbox{W}/\mbox{m}^{2}$) of a normalized source:

$$ E_{\mathit{band},\mathit{norm}} = \int _{0.25~\upmu \text{m}}^{1.1~\upmu \text{m}} E _{\mathit{norm}} (\lambda )* T_{\mathit{filter}} ( \lambda ) *d\lambda $$

Assuming that the source is Lambertian, we calculate the in-band radiance, $L _{\mathit{band}}$ ($\mbox{W}/\mbox{m}^{2}/\mbox{sr}$), of the source by dividing the in-band irradiance by the solid angle, $\varOmega _{\mathit{det}}$ (sr), of the calibrated detector.

$$ L_{\mathit{band}} = \frac{E_{\mathit{band}}}{\varOmega _{\mathit{det}}} $$

To calculate the responsivity of the camera, $R$ [$\mbox{DN}/\mbox{s}/(\mbox{W}/\mbox{m}^{2}/\mbox{sr})$], we divide the camera signal, $S _{DN}$, by the effective exposure time (Sect. 2.4.2) of the image, $t_{\mathit{exp}}$, and the in-band radiance. To calculate spectral responsivity [$\mbox{DN}/\mbox{s}/(\mbox{W}/\mbox{m}^{2}/\mbox{sr}/\upmu \mbox{m})$] we also multiply by the bandwidth of the filter, $T _{bw}$.

$$ R= \frac{S_{DN}}{t_{\mathit{exp}} L_{\mathit{band}}}\qquad R_{\mathit{spec}} = \frac{S_{DN}}{t_{\mathit{exp}} L _{\mathit{band}}} T_{bw} $$

The filter bandwidth is defined as the integral under the filter transmission curve (Rizk et al. 2018).

$$ T_{bw} = \int _{0.25~\upmu \text{m}}^{1.1~\upmu \text{m}} T_{\mathit{filter}} ( \lambda ) d \lambda $$

To apply the radiometric calibration, the calibration pipeline converts camera signal, $S_{\mathit{obs}}$, to physical units of radiance or spectral radiance, $L_{\mathit{obs}}$, by dividing the measured signal by the exposure time, $t_{\mathit{exp}}$, and the calibrated responsivity, $R$.

$$ L_{\mathit{obs}} = \frac{S_{\mathit{obs}}}{t_{\mathit{exp}} R} $$

In addition to radiance, the pipeline also produces OCAMS images in units of reflectance factor. Reflectance factor is an ambiguous term in the literature and is often used interchangeably with radiance factor and I/F. Here we take the standard definition that reflectance factor is the dimensionless ratio of reflected light from a target to reflected light from a Lambertian disk (Li et al. 2015). Reflectance factor is calculated as a function of distance to the Sun and the in-band solar flux at 1 AU.

$$ \frac{I}{F} = L_{\mathit{obs}} *\pi * \frac{D_{\mathit{sun}}^{2}}{F_{\mathit{band}}} $$

where $D_{\mathit{sun}}$ is the distance from the Sun to the surface (AU) and $F _{\mathit{band}}$ is the in-band solar flux ($\mbox{W}/\mbox{m}^{2}$) at 1 AU. Because we correlate the solar flux with the radiance formulation, we calculate it over the bandwidth of the filter; the OCAMS values for solar flux are given in Table 3.

**Table 3:** Effective center wavelengths, cut-on/off wavelengths, and solar flux values of OCAMS filters

Camera/filter	Effective center wavelength (nm)	Filter cut-on/off wavelengths (nm)	In-band solar flux (W/m^2^)	In-band spectral solar flux (W/m^2^/μm)
MapCam/b′	473	439–500	–	2003.2
MapCam/v	550	521–578	–	1837.8
MapCam/w	698	671–731	–	1426.9
MapCam/x	847	815–893	–	993.8
MapCam/pan^†^	646	489–815	501.0	–
PolyCam/pan	650	482–808	490.6	–
SamCam/pan^†^	644	488–813	504.3	–

^†^Mean of pan filters

#### Temperature Dependence

**Fig. 10:**
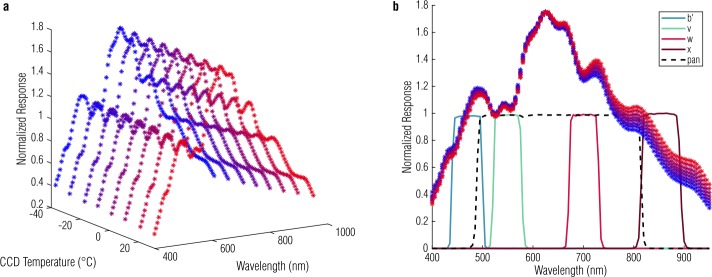
Temperature-spectral responsivity as measured in a flight-spare detector. Data points are colored from red to blue to represent their temperatures from hot to cold. The bandpasses of the b′, v, w, and x filters are overlaid in blue, green, red, and dark red from left to right; the panchromatic filter is indicated with a dashed black line. There is no significance to the relative height of the pass bands and the normalized detector response; they are scaled for display

As with all silicon-based detectors, the responsivity of the OCAMS CCDs is thermally dependent, and that dependence is coupled with wavelength (Sato et al. 2013). In general, CCDs are more sensitive to longer optical wavelengths (e.g., 900 nm) when they are warmer and less sensitive at shorter wavelengths (e.g., 400 nm), though the effect is strongest at long wavelengths. We measured the responsivities of the OCAMS cameras at room temperature during ground testing, but we acquire most flight data at colder temperatures (${\sim} {-}20\ ^{\circ}\mbox{C}$). To characterize the full range of temperature dependence, we measure the responsivity of a flight-spare detector (made from the same wafer as the detectors in the cameras) as a function of both wavelength and temperature. Figure 10 shows the data from that test, where the data points are color-coded from red to blue to indicate hot to cold, respectively. The plot shows an increase in detector responsivity at higher temperatures. For reference, the plot on the right overlays the bandpasses of the OCAMS filters.

Analysis of these data and data taken during thermal-vacuum ground testing with the flight cameras produces a linear temperature dependence for each filter. When the calibration pipeline converts OCAMS images to radiance, it first adjusts the responsivity term for this dependence, listed in Table 4.

$$\begin{aligned} \mathit{tsr} ' =& \bigl( 1+ ( T_{ccd} - T_{\mathit{ref}} ) *\mathit{tsr} \bigr) \\ R ' =&R*\mathit{tsr} ' \end{aligned}$$

where $T_{\mathit{ccd}}$ is the temperature of the CCD, $T_{ \mathit{ref}}$ is the temperature at which the nominal responsivity was measured, $\mathit{tsr}$ is the slope of the temperature-spectral responsivity dependence, $R$ is the nominal responsivity for that filter at the reference temperature, and $R'$ is the adjusted responsivity. See Sect. 3.3 for a discussion of the nominal responsivity values.

**Table 4:** Thermal-spectral responsivity dependence

Camera/filter	Thermal-spectral responsivity slope (^∘^C^−1^)	Reference temperature (^∘^C)
MapCam/b′	−0.0014	30.2
MapCam/v	−0.00075	30.0
MapCam/w	0.00053	30.1
MapCam/x	0.003	26.6
MapCam/pan	0.00075	28.6
PolyCam/pan	0.00075	27.2
SamCam/pan	0.00075	29.6

#### Detector Linearity

Linearity is a measure of a detector’s gain as a function of signal level and is a common diagnostic of a detector’s performance. Detector gain, the DN recorded per electron generated (Janesick 2001), is approximately 4.5 DN/e^−^ for the OCAMS CCDs (Rizk et al. 2018). For an ideal detector, the gain is independent of the signal level; one of the advantages of CCDs is their generally high level of linearity. Nonetheless, it is expected that even CCDs will have some amount of nonlinearity at very low and very high signal levels, with the latter occurring as the detectors approach saturation (Downing et al. 2006; Gosset and Magain 1993; Janesick 2001). We have investigated the linearity of the OCAMS detectors by examining data from ground testing with the engineering and flight models of the cameras. Because a complete linearity test was not possible given the schedule for the flight camera ground testing campaign, we expanded upon that data with a specialized linearity test with a flight-spare detector after launch.

**Fig. 11:**
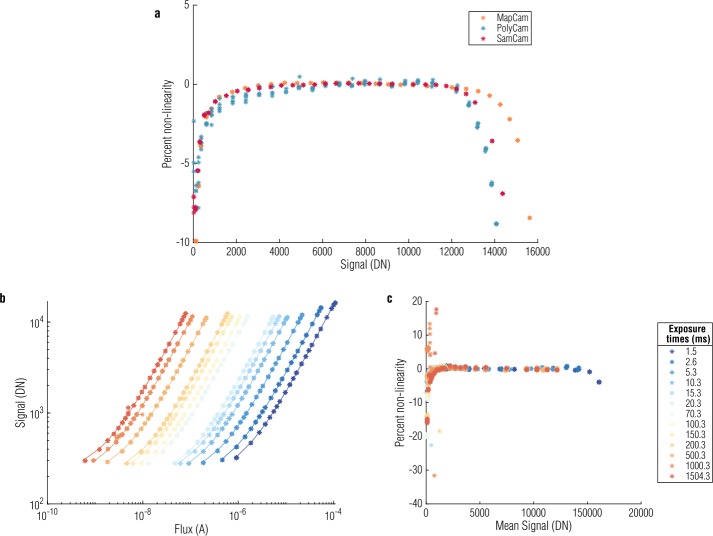
Measuring linearity with the flight cameras (**a**) identifies the linear regime. A more comprehensive test with a flight-spare detector at a series of exposure times (**b**), where color indicates a unique exposure time, provides a large data set that covers the range of light levels expected at Bennu. Independently analyzing each exposure time produces a summary of detector linearity across this dynamic range (**c**) and confirms the results of the flight camera tests. The detectors are ≥2% nonlinear at signals below 1000 DN and above 14000, 12500, and 13000 DN for MapCam, PolyCam, and SamCam, respectively

Figure 11(a) plots linearity measured in radiance testing for the flight cameras. To estimate linearity, we calculate the ratio of signal measured to incident light (i.e., responsivity), normalized to the mean camera responsivity, as a function of signal level. A perfectly linear detector would have a flat response (i.e., the same responsivity at every signal level). Deviations from unity are represented in the figure as percent nonlinearity. For the flight cameras, linearity was performed at a single illumination level that allowed us to test the detector over a broad range of exposure times (0–2000 ms).

During post-launch testing, the flight-spare detector captured images at a single exposure time for a series of illumination levels. This was repeated at a series of exposure times, generating a large data set with a wide range of exposure times and light levels, as shown in Fig. 11(b). We measure linearity for each exposure time set independently to evaluate the linearity of the detector over the range of light levels expected throughout the mission. Figure 11(c) plots the deviation from linearity as a function of signal level. The results are in qualitative agreement with the linearity test of the flight units, indicating that the flight spares are a suitable analog for the OCAMS cameras in this investigation.

We do not correct for nonlinearity in the calibration pipeline. Though an exponential dependence appears present from Fig. 11(c), there is also considerable scatter at low signal levels. As a result, we have not identified a correction that we could apply with the precision required to systematically linearize data at low signal levels. Hence, for scientific analyses of OCAMS data, we define a canonical ‘linear’ signal range using the central portion of the dynamic range. The definition of acceptable linearity is subtle and guided by the scientific intent of the data under consideration. The low end, in particular, changes slowly, leading to a gradual cut-off with the linear regime. Signals that are above 2000 DN, 1000 DN, and 500 DN are linear to the 99.5%, 99%, and 98% levels, respectively. The high end of the dynamic range has a sharper cut-off and, as shown in Fig. 11(a), is specific to the detector: 14000 DN for MapCam, 12500 DN for PolyCam, and 13000 DN for SamCam. OCAMS data that are intended for spectrophotometric analyses are designed to achieve signal levels that fall within the 99.5% linear regime. The corresponding nonlinearity can be propagated forward into scientific analyses that rely on values of absolute radiance or reflectance. The non-linearity threshold, as well as the saturation threshold of 16383 DN, are documented in the FITS header of every image in the LINLIM and SATLIM keywords, respectively. The thresholds are given in the relevant units for an image (radiance, spectral radiance, or reflectance).

### Charge Transfer Efficiency

Charge transfer efficiency (CTE) is a standard metric for CCDs that measures their ability to transfer charge between potential wells (pixels). That is, some trailing charge is left behind with each charge transfer. As such, pixels that are further from readout will leave more charge behind as they are transferred off the array. Modern scientific CCDs have CTE in the vertical transfer direction $\geq 0.99999$ and CTE in the horizontal direction $\geq 0.999999$ (Janesick 2001). A vertical CTE of 0.99999 for a $1024\times1024$-pixel array will result a worst case of 1% lost signal for the furthest pixel. Vertical CTE was measured during the ground testing campaign and is monitored periodically in flight. Ground and flight tests use the extended pixel edge response (EPER) method, wherein the detectors are exposed to the internal calibration sources and the rows are transferred backward (away from the readout, i.e., down in Fig. 3) to expel charge from the rows furthest from readout. The array is then read out as normal; the rows furthest from readout include only trailing signal from the rows between them and readout (Mutchler and Sirianni 2005). The magnitude of the trailing charge is a direct measure of the CTE. To date, the vertical CTE values for various signal levels as a percentage of full well (FW) have not measurably changed (Table 5) and remain $> 0.99999$.

**Table 5:** OCAMS vertical charge transfer efficiencies

Camera	CTE at 20% FW	CTE at 40% FW	CTE at 65% FW
MapCam	0.9999914	0.9999958	0.9999977
PolyCam	0.9999913	0.9999955	0.9999972
SamCam	0.9999886	0.9999946	0.9999965

## In-Flight Calibrations

In-flight calibration campaigns have two primary goals: to track the performance of the cameras from their known pre-flight condition through the encounter with the asteroid and to evaluate aspects of camera calibration that we can perform more accurately in flight than on the ground. The pre-launch plan for these campaigns is described in Rizk et al. (2018) and updated in this manuscript. Specifically, we discuss monitoring RTS pixels, measuring geometric distortion and stray light, and updating the ground-based radiometric conversions with lunar data from OSIRIS-REx’s EGA (Golish et al. 2018).

### Dark Current and RTS Pixels

Exposure to radiation during flight has changed the character of the dark current in the OCAMS detectors. While the overall dark current generation rate is stable, the population of pixels exhibiting both dramatically increased responsivity (hot pixels) and multi-stable responsivity (RTS pixels) has increased. Bad pixels maps are updated with the increased census of both of these populations throughout flight.

Because of its variable nature, RTS pixel behavior is less well captured by a bad pixel map. Before launch, RTS manifested as individual pixels that changed behavior (from hot to normal and back again) over time. After exposure to radiation, this behavior increased and expanded to include regions of (rather than individual) pixels that have variable behavior. This is most evident in long-exposure (>10 seconds) images that record star fields and the initial acquisition and survey of Bennu (Hergenrother et al. 2019). Correcting dark current is critical for analysis of long-exposure images, but RTS regions can corrupt a master dark by elevating the dark signal in those pixel regions. When those master darks are then applied to images where the RTS region is not as sensitive, the resulting image will appear to have ‘holes’ where the RTS regions were over-corrected.

This behavior prompted two changes in our standard operating procedure. The first is to prefer stacking multiple lower-exposure images together, rather than taking one long-exposure image, to achieve a desired signal-to-noise ratio. For example, three 10-second images will see nearly as faintly as a single 30-second image, but will be less susceptible to RTS regions. Shorter images are also less susceptible to spacecraft drift during imaging. Multiple shorter exposures also allow us to eliminate cosmic rays from image data by combining the images with a median operation.

The second change is a more careful evaluation of RTS regions and subsequent generation of bad pixel masks targeted for specific observations. Rather than having a mission-wide bad pixel map that tracks the historically aberrant pixels, targeted bad pixel maps identify the relevant bad pixels for a given observation. We create and archive these masks with the corresponding master darks (Rizk et al. 2019).

### Flat Field

**Fig. 12:**
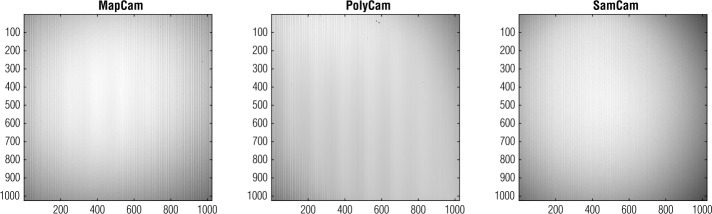
Calibration lamp images from the 6-month post-launch checkout for each camera. We will monitor changes in these images from before launch and throughout the mission to update the master flats as necessary

Radiation can also affect the sensitivity of the OCAMS detectors to optical light. The ground-based master flats capture the optical vignetting (which has not changed in flight) and the responsivity of individual pixels. During pre-launch ground testing, we created flat fields by observing a spatially invariant source (Rizk et al. 2018; Rizk 2001) with the cameras, which is not available in flight. Instead, each camera has an on-board illumination system (calibration lamp) that allows us to update the master flats (Rizk et al. 2018). We cannot directly create a master flat from the lamp images because the illumination patterns are not uniform, as shown in Fig. 12. However, we can track changes in the calibration lamp images throughout the mission and update the responsivity of individual pixels correspondingly. To date, the on-board lamp images have demonstrated no statistically significant change in the responsivity of the OCAMS detectors. We will acquire lamp images throughout the rest of the mission and will apply flat field corrections as necessary.

### Aliasing

The OCAMS detectors, as with many CCDs (Murchie et al. 1999; Sierks et al. 2011), do not have 100% fill factor on their pixels due to electronic structures on and below the surface of the silicon (Sect. 1.2). For the vast majority of OCAMS observations, which will be of extended objects, the insensitive areas are not relevant. The radiometric calibration of the detectors was done by measuring the response of the detector to a known source (Sect. 2.6) and therefore inherently includes the true fill factor of the pixels. However, the impact of these insensitive areas is felt strongly when observing point sources. The point spread function (PSF) of the cameras is on the order of a pixel, such that the location of the image of a point source on the detector, relative to the insensitive areas, can have a dramatic impact on the fraction of that point source that is detected. We refer to this phenomenon as *aliasing*.

The effective shape of the system PSF is a convolution of the optical PSF and the step functions that describe the sensitive areas of the pixels. Ideally, we would be able to calculate that PSF in order to analytically predict the amount of light detected by the pixels for a given optical PSF. However, the exact form of the detector geometry is unknown. This is true for the size and locations of the masked regions, which will have some device-level variation, but also for the opacity of the regions themselves, which may be wavelength-dependent.

**Fig. 13:**
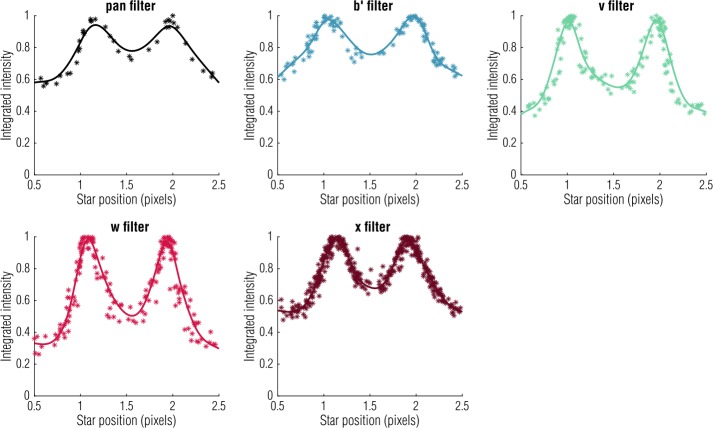
Plotting intensity of a point source as a function of pixel position demonstrates a different aliasing pattern for each MapCam filter and suggests a potential correction. However, independently acquired data (of other point sources or in different regions of the detector) do not follow the same pattern. Therefore, the OCAMS calibration pipeline does not include an automatic correction for this effect

Due to these ambiguities, we have performed in-flight observations to characterize the aliasing effect. During these observations, MapCam acquired images while a star field drifted across the field of view, such that the point sources crossed several pixel column boundaries. This allowed us to plot the integrated intensity of the point sources as a function of their horizontal position, as shown in Fig. 13. However, repeating this calibration for other regions of the detector (where the optical PSF is slightly different) or at different times (when slight changes in detector temperature also change the PSF width) does not produce consistent results. As such, the calibration pipeline does not currently include a correction for this effect. The number of observations for which it was necessary was limited to the Approach phase of the mission (spanning approximately 2 months) when Bennu was a point source. These data must be treated differently in any post-processing. The aliasing calibration observations, performed in March and August of 2018, are archived in the PDS Small Bodies Node with OCAMS flight data (Rizk et al. 2019).

### Radiometry

**Fig. 14:**
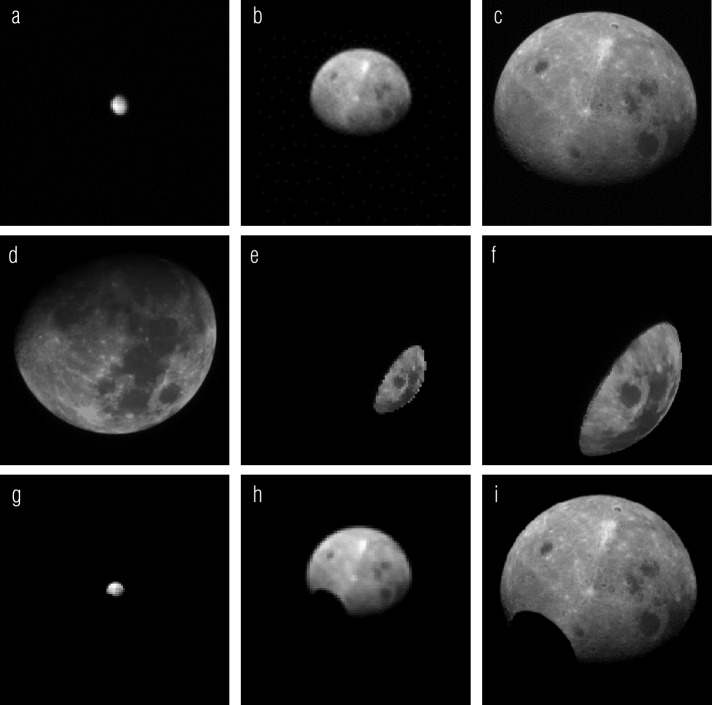
SamCam, MapCam, and PolyCam images of the Moon during OSIRIS-REx’s EGA (**a**–**c**). Comparison of the EGA images with a ROLO image (**d**) re-projected to match OCAMS geometry (**e**, **f**) updates the radiometric calibration of the cameras. Comparison with simulated images using a Kaguya MI photometric model of the Moon (**g**–**i**) verifies the calibration for MapCam and PolyCam and directly provides the calibration for SamCam

We developed the radiometric calibration (the conversion from signal in DN to physical units of radiance or I/F) for the cameras during ground testing with calibrated laboratory sources. However, these calibrations are vulnerable to common laboratory errors, including imperfect source calibration and stray light. In flight, we had the opportunity to update our radiometric calibration with lunar observations acquired during EGA. The EGA images are of particular value because the Moon is a well-studied, temporally invariant, extended target. Figure 14(a–c) shows SamCam, MapCam, and PolyCam images of the Moon taken during EGA. MapCam acquired images in all five bands.

**Fig. 15:**
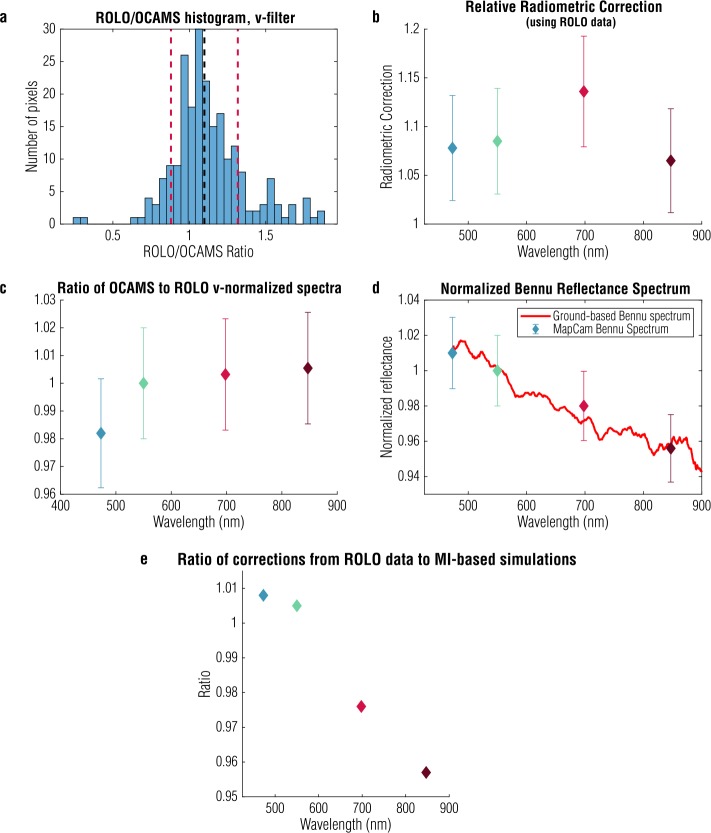
Taking the ratio of OCAMS data to ROLO data taken at the same phase angle produces a histogram (**a**); excluding data outside two standard deviations (red dashed lines), we calculate the mean (black dashed line) to find the radiometric correction applied to the ground calibration. The MapCam filters required corrections between 6 and 14% (**b**). Calculations of band ratios on ROLO and OCAMS data separately (**c**) and a comparison of MapCam measurements of Bennu to ground-based observations (**d**) demonstrate a <2% relative radiometric uncertainty. Comparison with simulations based on a Kaguya Multiband Imager basemap independently determines an absolute radiometric uncertainty of ∼5% (**e**)

We compare these data to images taken by the RObotic Lunar Observatory (ROLO) (Kieffer and Stone 2005). Figure 14(d) shows a ROLO image taken at the same phase angle (${\sim} 42^{\circ}$) as the OCAMS EGA images. We use the ROLO wavebands that best match the OCAMS filters (Table 3): 475 nm for b′, 553 nm for v, 703 nm for w, 865 nm for x, and 665 nm for pan. Because OCAMS was partially behind the moon during EGA, and ROLO observes the Moon from Earth, only a subset of the lunar surface is in common with the OCAMS images. Nonetheless, enough of the surface is viewed by both systems to perform this calibration. We re-project the ROLO data to OCAMS image space and reduce the data to the common subset, shown in Fig. 14(e–f). We also apply a McEwen photometric correction developed for the Moon (McEwen 1996) to account for the differences in observation geometry. This correction is imperfect, particularly on the limb, so we remove outliers that are more than two standard deviations away from the mean of the ensemble. Finally, we calculate the ratio of the OCAMS and ROLO images and produce a histogram. We show an example for the MapCam v filter in Fig. 15(a); the other filters have similar behavior. The mean of the distribution is the scalar correction we apply to our radiometric correction (i.e., to match the mean OCAMS reflectance to the ROLO reference). Figure 15(b) shows the correction calculated for each filter, as a function of wavelength, and indicates that the reflectance values measured by MapCam were between 6 and 14% too low.

The standard deviations of the distributions are high, due in part to the projection and photometric errors introduced by this process, as well as the relatively small number of pixels in common. To investigate how this lack of precision affects the radiometric uncertainty, we consider the absolute and relative accuracy independently.

For much of the color processing anticipated for the mission, such as color and color ratio maps used to characterize the composition of the surface and identify regions of interest (DellaGiustina et al. 2018) and regional photometric analyses, the absolute radiometric calibration of the cameras is less critical than the relative filter-to-filter calibration. Fortunately, the scatter in the ROLO/OCAMS calibration largely does not affect the relative calibration. We can confirm this by calculating a lunar spectrum normalized to the v band for both ROLO and MapCam data, after updating its calibration. That is, we divide each OCAMS or ROLO image by the corresponding v-band image (553-nm band for ROLO). Performing this division in camera space, rather than after projection or photometric correction, eliminates the noise introduced by those operations. Assuming that the v-normalized spectrum is similar across the Moon’s surface, we can also use the entire disk of the Moon seen in each image, rather than just the common subset of the surface observed by both OCAMS and ROLO. We then calculate the mean of the result, ignoring pixels that have a reflectance less than 0.005 (to mask out off-body pixels). We plot the means in Fig. 15(c), which shows less than 2% variation between the v-normalized spectra.

We further validate the relative radiometric accuracy by comparing a MapCam v-normalized spectrum of Bennu to ground-based measurements (Clark et al. 2011), as shown in Fig. 15(d). The ground-based Bennu data did not directly determine the OCAMS calibration, but they do demonstrate <1% error in our relative calibration. Based on these two validations, we assign a 2% relative radiometric uncertainty for the MapCam color filters.

For other processing tasks, such as producing filter-specific albedo maps of the surface and calculating the corresponding albedo distribution (Lauretta et al. 2019), the absolute radiometric uncertainty is paramount. To assess the absolute radiometric accuracy, we perform an independent calibration with an illumination model of the Moon from NASA Goddard Space Flight Center (Mazarico et al. 2018), based on SELENE (Kaguya) Multiband Imager (MI) global mosaics (Lemelin et al. 2019; Ohtake et al. 2013). We simulate images of identical observation geometry, as shown in Fig. 14(g–i) at the MapCam filters’ effective wavelengths. This has the advantage of using the majority of the surface imaged by OCAMS (except for a small portion not detailed by the underlying albedo basemap). Topographic corrections to the viewing and illumination geometry are based on a combined SELENE/Lunar Orbiter Laser Altimeter lunar shape model (Barker et al. 2016). The hole in the lower-left region, which we exclude from the analysis, is due to missing data in the albedo map upon which the simulation is based. As with the ROLO analysis, we filter out pixels that are further than two standard deviations from the mean of the whole disk. Figure 15(e) plots the ratio of the whole disk mean (with outliers removed) for the ROLO- and MI-based images, by MapCam filter, and shows that they agree with each other to within 5%. We also generated a set of simulated OCAMS images based on Lunar Reconnaissance Orbiter Camera (LROC) Wide-Angle Camera (WAC) global mosaics (Sato et al. 2014) and found that they agreed with the MI-based images to within 2% for the b′, v, and w filters. The WAC radiometric calibration is tied to ROLO data, but only covers wavelengths 321 to 689 nm (Mahanti et al. 2016). The absolute radiometric accuracy of the ROLO data and MI-based simulations is estimated at 5 to 10% (Ohtake et al. 2010; Stone and Kieffer 2004) but agrees with the ROLO-based calibration to better than 5%. We therefore assign a 5% absolute radiometric accuracy to the OCAMS images.

We selected the ROLO-based calibration for MapCam and PolyCam because the MI bands do not match OCAMS as closely as the ROLO bands. Because the Moon is so small in SamCam’s field of view, made even smaller by the amount of overlap between the ROLO and OCAMS images, we do not apply the ROLO-based calibration method to SamCam. However, the close agreement of MI and ROLO is encouraging and allows us to use the MI-based calibration for SamCam. Moreover, SamCam’s primary purpose is to document acquisition of a sample (Rizk et al. 2018), so this instrument has less strict radiometric requirements.

We modify the radiometric correction constants in the OCAMS calibration pipeline to the values given in Table 6, which represent an update to the ROLO calibration values presented in DellaGiustina et al. (2019). These values are given at the reference temperatures listed in Table 4; temperature corrections to responsivity must still be applied as described in Sect. 2.6.1.

**Table 6:** Updated OCAMS radiometric conversion constants

Camera/filter	Radiometric conversion constant [DN/s/(W/m^2^/sr)]	Spectral radiometric conversion constant [DN/s/(W/m^2^/sr/μm)]
MapCam/b′	–	22900
MapCam/v	–	29900
MapCam/w	–	52900
MapCam/x	–	51900
MapCam/pan^†^	761000	–
PolyCam/pan	556000	–
SamCam/pan^†^	257000	–

^†^Mean of pan filters

### Stray Light

The stray light performance of the cameras was measured as part of ground testing (Rizk et al. 2018), which showed excellent rejection of both in-field and out-of-field sources of stray light at expected levels. However, the models and laboratory tests were based on the camera structures themselves and did not consider sources of stray light from the rest of the spacecraft. Images from in-flight system health checkouts revealed that the cameras are susceptible to stray light reflecting off other instruments and structures on the spacecraft in some illumination geometries. Subsequent analysis using a more comprehensive stray light model of the entire spacecraft is in good agreement with observations. That analysis has allowed us to identify spacecraft orientations (with respect to the Sun) that are more favorable for minimizing stray light for key observations throughout the mission. Those predictions were then tested in flight by taking images with the spacecraft positioned in a range of orientations around the expected optimal conditions.

This combination of model, observation, and operational constraints has been used to completely mitigate the stray light in PolyCam observations. It has also been very successful for MapCam imaging campaigns in the Detailed Survey phase of the mission (Lauretta et al. 2017). The campaign in search of dust plumes during Detailed Survey combined long exposures and unfavorable observation geometries (${\sim} 130^{\circ}$ phase) where the Sun illuminated the science deck (Lauretta et al. 2017). Operational constraints during this phase of the mission prevented us from exploiting the more favorable spacecraft orientations identified prior to the spacecraft’s arrival at Bennu. Working within the operational and planning constraints for this phase, we were able to acquire some deep space images away from Bennu, in a similar solar configuration. These images are used to understand and model the properties of solar stray light observed during the plume search campaign. In addition to stray light from the Sun, images from this campaign were subject to an equal amount of stray light from Bennu itself. During the plume search, the camera followed a circle around the crescent asteroid multiple times while Bennu completed a full rotation. This circle was slightly offset such that the camera imaged Bennu’s dark limb but missed its bright limb. The result was a spatially extended stray light source of varying intensity and direction (due to Bennu’s irregular shape located just outside of MapCam’s field of view, which is difficult to model). However, we were able to take advantage of the spatial characteristics of the stray light and of methods developed to model the solar stray light to process the plume search data.

The sampling event (which will include bright reflections from the head of the Touch-And-Go Sampling Acquisition Mechanism) presents a challenging stray light environment that was carefully analyzed during the development of OCAMS (Choi et al. 2019; Choi 2016; Lauretta et al. 2017). In comparison, the spacecraft-level stray light discovered after launch is expected to be relatively benign for sampling, but the actual impact will depend on the exact latitude of the sampling site and the precise flight path. The stray light model informs observation designs to minimize the impact of stray light and allows us to quantify the expected impact when operational constraints limit those designs. A complete discussion of the observatory-level stray light model, in-flight testing, analysis of the plume search observations, and impact on observation design is outside the scope of this manuscript.

### Geometric Distortion

Correction for geometric distortion in OCAMS images is critical both for optical navigation of the spacecraft (Pelgrift et al. 2018) and high-fidelity mosaicking of images during the mapping and reconnaissance phases of the mission (DellaGiustina et al. 2018). Observations of star fields during flight have provided a thorough dataset with which to calculate the distortion (Pelgrift et al. 2018). For PolyCam, ground test data is also available at several focus positions between 200 m and infinity. Our distortion model varies as a function of focus position, following a formalism derived from the camera’s optical model. The model is bounded on one end by the distortion model derived from stellar observations (also used by the navigation team) and on the other end by the distortion derived at 200 m from the pre-flight testing. This PolyCam distortion model supersedes the one presented in Pelgrift et al. (2018).

We have incorporated the results of this work, in the form of distortion models, into the ISIS3 (Integrated Software for Imagers and Spectrometers, version 3; U.S. Geological Survey) camera models that are used to geospatially register images from MapCam and PolyCam. The software includes distortion parameters for all 93 PolyCam focus positions; Table 7a presents a subset at the most commonly used ranges. Distortion in MapCam does not meaningfully change from filter to filter, but the camera’s focal length does. The effective focal lengths for each filter are given in Table 7b and the distortion parameters are shown in Table 7c. For both cameras, the radial distortion $\Delta \rho $ (in millimeters), at any location in the image, is calculated with the following formula.

$$ \Delta \rho = p_{1} \rho + p_{2} \rho ^{2} + p_{3} \rho ^{3} $$

where $\rho $ is the distance from the center of distortion, in millimeters. The ideal undistorted position of an object is shifted away from the center of distortion by $\Delta \rho $. In this convention, a positive $\Delta \rho $ corresponds to pin cushion distortion and a negative $\Delta \rho $ corresponds to barrel distortion. We compensate for distortion simultaneously with map-projecting images for the creation of higher-level data products to reduce the number of times the image data is resampled (DellaGiustina et al. 2018). As such, the OCAMS calibration pipeline does not correct distortion.

**Table 7a:** PolyCam distortion model parameters

Focus distance	Motor position	Focal length (mm)	Center of distortion	P1	P2	P3
Infinity	17371	628.90	(512.2, 502.0)	0	6.37 × 10^−5^	3.16 × 10^−5^
5 km	16830	628.38	(511.6, 502.0)	0	6.07 × 10^−5^	3.18 × 10^−5^
3.7 km	16650	628.21	(511.5, 502.0)	0	5.97 × 10^−5^	3.19 × 10^−5^
700 m	13410	624.99	(509.1, 502.2)	0	4.29 × 10^−5^	3.33 × 10^−5^
500 m	11790	623.32	(506.8, 502.4)	0	3.49 × 10^−5^	3.40 × 10^−5^
225 m	5670	616.66	(500.4, 502.9)	0	6.73 × 10^−6^	3.66 × 10^−5^

**Table 7b:** MapCam focal lengths (mm)

pan	b′	v	w	x
125.2	125.37	125.11	125.13	125.42

**Table 7c:** MapCam distortion (pan)

Center of distortion	P1	P2	P3
(513, 513)	0	6.37 × 10^−5^	3.16 × 10^−5^

## Conclusions

The OCAMS performance verification campaign included a variety of calibrations that are applied throughout the OSIRIS-REx mission. All of these calibrations are integrated into the pipeline that automatically processes incoming OCAMS images (Selznick 2017).

Observations during cruise and images of the Moon during the EGA maneuver have allowed us to update many of the initial calibrations performed before launch. Of particular importance is the radiometric fidelity of the images with which we create mission-critical data products. These products require a clear understanding of the dark and flat field behavior of the cameras to remove those common sources of noise. Generation of data products without visual artifacts necessitates effective charge smear correction algorithms, which we have been able to test in realistic conditions. Finally, the radiometric conversion itself must be as accurate as possible, particularly for filter-to-filter comparison.

Radiometric accuracy is of particular importance for creating several scientific data products, including global albedo and color-ratio maps of Bennu, as well as analysis of individual features on the surface. An extensive analysis effort with ROLO data and illumination models of the Moon verify our absolute radiometric calibration to within 5% and our filter-to-filter calibration to an uncertainty of 2%.
